# Direct Visualisation of Skyrmion Lattice Defect Alignment at Grain Boundaries

**DOI:** 10.1186/s11671-022-03654-y

**Published:** 2022-01-28

**Authors:** Thomas Schönenberger, Ping Huang, Lawrence D. Brun, Li Guanghao, Arnaud Magrez, Fabrizio Carbone, Henrik M. Rønnow

**Affiliations:** 1 Laboratory for Quantum Magnetism (LQM), Institute of Physics, École Polytechnique Fédérale de Lausanne (EPFL), Lausanne, Switzerland; 2 State Key Laboratory for Mechanical Behavior of Materials, Xi’an Jiaotong University, Xi’an, China

**Keywords:** Lorentz transmission electron microscopy, Dislocations, Skyrmions, Defects, Grain boundaries

## Abstract

We present a method to directly visualise a statistical analysis of skyrmion defect alignment at grain boundaries in the skyrmion host $\hbox {Cu}_2$OSeO_3_. Using Lorentz transmission electron microscopy, we collected large data sets with several hundreds of frames containing skyrmion lattices with grain boundaries in them. To address the behaviour of strings of dislocations in these grain boundaries, we developed an algorithm to automatically extract and classify strings of dislocations separating the grains. This way we circumvent the problem of having to create configurations with well-defined relative grain orientations by performing a statistical analysis on a dynamically rearranging image sequence. With this statistical method, we are able to experimentally extract the relationship between grain boundary alignment and defect spacing and find an agreement with geometric expectations. The algorithms used can be extended to other types of lattices such as Abrikosov lattices or colloidal systems in optical microscopy.

**Supplementary Information:**

The online version contains supplementary material available at 10.1186/s11671-022-03654-y.

## Introduction

Skyrmions are swirling spin textures with a non-trivial topology and particle-like behaviour. Originally Tony Skyrme proposed what was going to be known as a skyrmion as a solitonic solution to describe baryons and mesons in particle physics [1]. The concept of skyrmions was shelved until its analogue in condensed matter systems was proposed in 1989 [2], and they were first observed experimentally in 2009 by Mühlbauer et al. [3]. Their discovery has triggered extensive research efforts due to their potential application in new spintronic devices. They range from new types of memory storage schemes [4] to microwave electronics [5], logic devices [6] to more exotic things such as probabilistic computing [7].

One of the most common appearances of skyrmions is in the form of a hexagonal lattice. There are two distinct ways of how to describe these lattices: either in the wave picture by considering the superposition of three q-vectors or in the particle picture. Although individual skyrmions have been found in various systems [8, 9], much until recently the question of how much of a particle-like behaviour skyrmions adopt in a lattice configuration was less clear. However, recent studies provide strong evidence to support a quasi-particle description in the lattice phase [10]. The particle-like behaviour of skyrmions offers the possibility to study lattices, defects and phase transitions in 2D. While the framework for the analysis of reciprocal space data is well established in various neutron scattering studies [11, 12], this is much less the case for real space data. This therefore calls for the development and implementation of suitable image processing algorithms and real space data analysis routines. We follow along these lines by looking at imperfect lattices and mismatched lattices to study the behaviour of defects at grain boundaries within the particle picture using transmission electron microscopy (TEM). Furthermore, we provide another example of how real space imaging of skyrmions can be used as a testbed to explore structural dynamics and order in condensed matter systems.

One of the most prominent representatives of skyrmion hosting materials is the insulating B20-type compound Cu_2_OSeO_3_. A wide range of experiments have been performed on this compound, ranging from small-angle neutron scattering (SANS) [13–15], AC susceptibility [16–18] to various Lorentz transmission electron microscopy (LTEM) studies [19, 20]. Furthermore, its magnetoelectric coupling allows to control its magnetic textures by external electric fields using the emerging electric polarisation as a handle. Recent experiments on electric field control pave the way for low power magnetic storage devices based on Cu_2_OSeO_3_ [21, 22]. Moreover, with its comparatively large helical pitch length of around $60\,\hbox {nm}$ skyrmions in this compound decouple efficiently from the atomic lattice and can hence form neat skyrmion lattices [3, 10]—ideal conditions to investigate the phenomena studied in this paper.

## Sample Preparation and Methods

We use cryo-Lorentz transmission electron microscopy (LTEM) in Fresnel mode to acquire a movie of a dynamically evolving skyrmion lattice configuration in real space and real time using thin slabs of a Cu_2_OSeO_3_ single crystal. LTEM is an ideal technique to acquire movies of such a system since it allows for large fields of views and relatively short acquisition times. Single crystals of Cu_2_OSeO_3_ were grown by chemical vapour transport redox reactions, aligned by Laue X-ray diffraction, and a slab with its main crystallographic axes along the [1 − 1 0], [− 1 − 1 2] and [1 1 1] directions was cut out of one of the single crystals. This slab was mirror polished and then used to prepare a high-quality TEM lamella oriented along the [111] direction with a thickness in the range of around $150\,\hbox {nm}$ (electron transparent at $300\,\hbox {kV}$) using the focused ion beam (FIB) microscope. FIB milling is a technique that allows to routinely produce large ($>10\,{\upmu \hbox {m}}$) and uniformly thick specimen. We finish the FIB preparation of the lamella by using a low-voltage ($5\,\hbox {kV}$) cleaning step to reduce the amorphous layer created by Ga-ion implantation. An O_2_-plasma cleaning procedure helps to remove organic contamination on the lamella surface. The TEM lamella is attached to a standard Omniprobe Cu TEM grid, and a thin carbon layer (few nms) is evaporated on the finished lamella to reduce charging effects in the TEM. Observations are carried out on an FEI Titan Themis TEM at 300 kV in Lorentz mode meaning a dedicated mini objective lens that is placed below the actual objective lens is used to produce the image. In this configuration, the objective lens is switched off to provide a magnetic-field free environment since it typically generates around $2\,\hbox {T}$ of magnetic field and would completely saturate most magnetic samples. It can, however, be used to apply any desired magnetic field perpendicular to the sample between zero and the maximum excitation by tuning the current in the lens. To see the magnetic contrast that is generated by the Lorentz deflection at the in-plane components of a magnetic sample, a defocus of up to $1\,\hbox {mm}$ is used (Fresnel imaging mode). Since the magnetic ordering temperature of Cu_2_OSeO_3_ is below $60\,\hbox {K}$ we cool down the sample using a Gatan liquid helium holder. Stable skyrmion lattice configurations are observed at about $30\,\hbox {K}$ after field cooling at $640\,\hbox {Oe}$. We acquire a movie for several minutes with an exposure time of $336\,\hbox {ms}$ at a resolution of 4096$\times$4096 pixels while maintaining a constant field and temperature. To analyse hundreds of frames each containing around 13,000 skyrmions, we have written custom MATLAB codes to analyse all TEM images.

## Results and Discussion

**Fig. 1:**
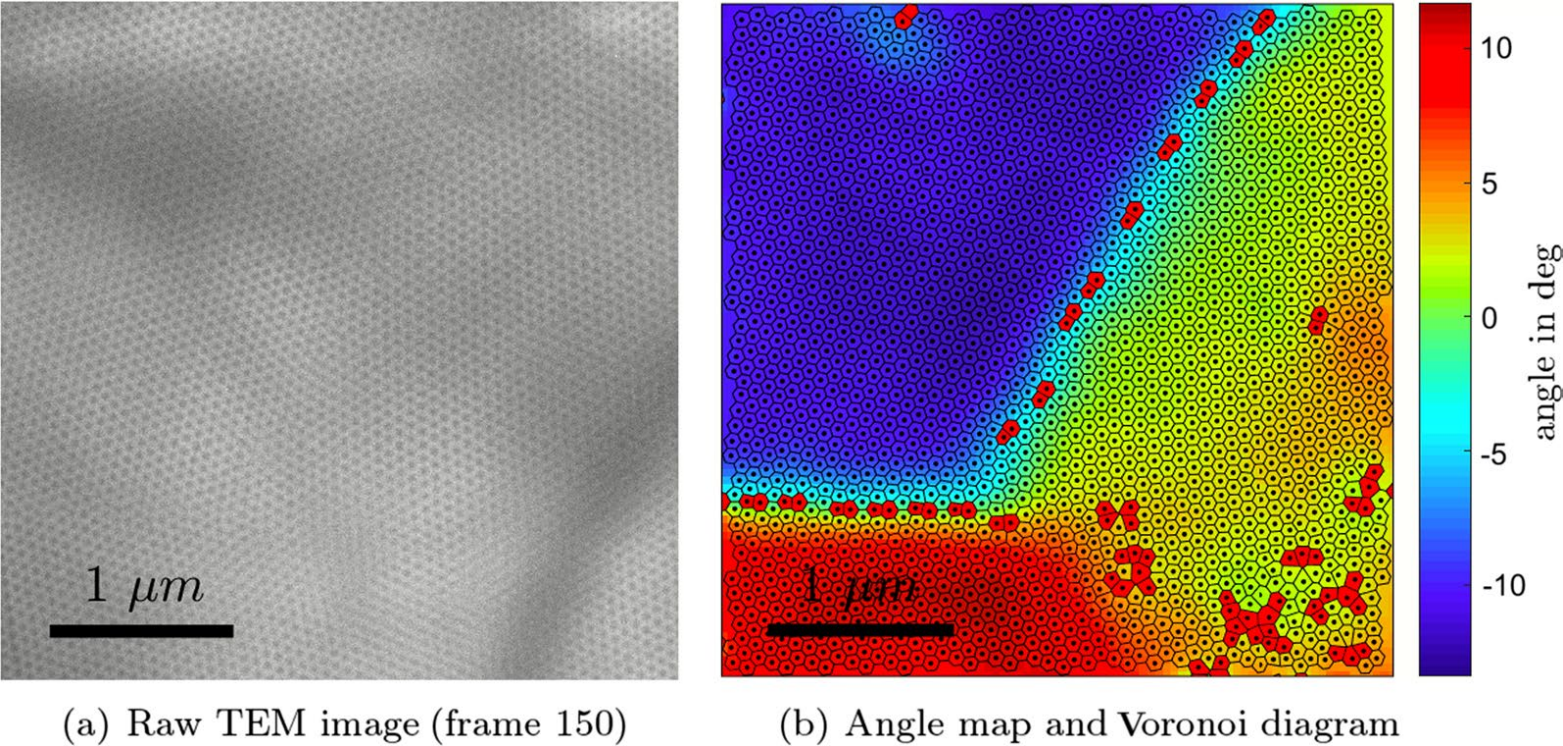
Example of one TEM micrograph from the series of 579 TEM frames analysed in this study (**a**) and the angle map revealing the different lattice domains (**b**). The identified points are used to construct the Voronoi diagram (**b**). Transparent polygons are sixfold coordinated sites, red polygons are defects

The skyrmion configurations are subject to fluctuations due to temperature and heat gradients created by the electron beam irradiation. As a result, the grain boundaries between regions of different lattice orientation change over time as well. This allows us to obtain grain boundaries separating a wide range of angles by extracting defect strings for each frame of the acquired TEM movie. In order to carry out the analysis, the position of each skyrmion for all the frames in the movie must be determined. To this end, we have developed a dedicated identification routine, which includes multiple correction steps that allow for a reliable identification even in blurry microscope images. Steps include the application of a log-type filter to smoothen the image and enhance contrast and the identification of local minima in image intensity. These local minima can in principle be identified as skyrmion positions if the intensity is below a certain threshold (determined by inspecting the result visually). More details can be found in Additional file 1. An exemplary TEM micrograph is shown in Fig. 1a along with the identified skyrmion positions together with the corresponding Voronoi tessellation in Fig. 1b.

The latter is a construction of polygons that encompass points that are closer to a particular skyrmion position than any other position. The edge-sharing polygons therefore yield information about the number of nearest neighbours of each skyrmion. Transparent polygons in Fig. 1b mark regular sixfold coordinated sites, whereas red polygons represent all non-sixfold coordinated sites hereafter referred to as defects. The most common defects are 5- and 7-defects that in the solid phase often appear as 5–7 pairs, known as dislocations. Note that while the Voronoi construction is precise, the presence of a defect can also just be an artefact. This is when the point identification is not precise, which can happen when during the acquisition time skyrmions rearrange and parts of the TEM image blur out (motion blur).

### Extracting Dislocation Strings

**Fig. 2:**
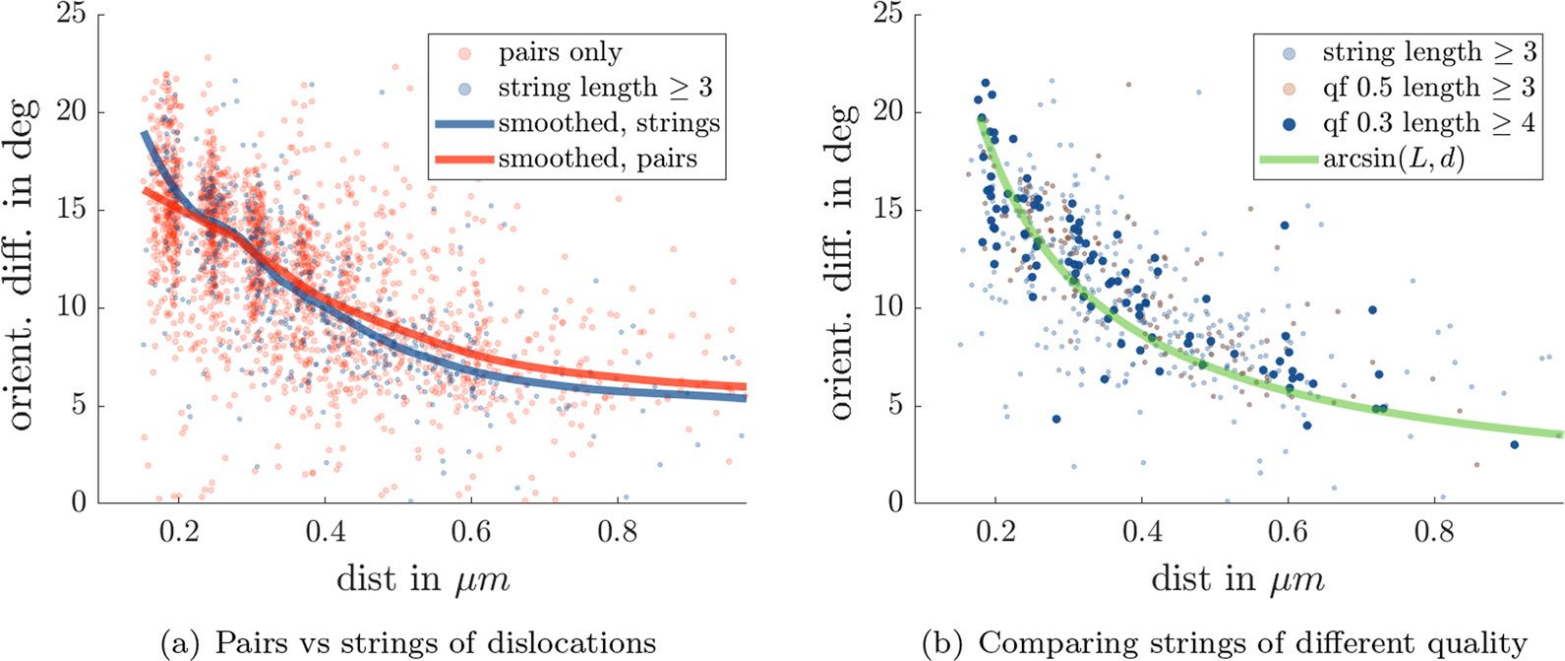
Comparing the difference in orientation vs median distance in the corresponding strings based on strings with a length of at least 3 (blue) and pairs of dislocations (red) (**a**). Comparing the difference in orientation vs median distance in the corresponding strings of different minimum lengths and quality factors (**b**)

We calculate the local orientation of the lattice based on the Voronoi diagram to yield angles between $-15^\circ$ and $15^\circ$ as shown in Fig. 1b. This way, the different domains are clearly revealed, and we see how they are separated by strings of dislocations (defect pairs). Our goal is to extract these defect lines in order to determine how the spacing of dislocations is related to the relative orientations of the adjacent regions. Since a controlled twisting of lattice orientations is hard to achieve, we study an entire movie and tackle the problem from a statistical point of view. Based on the angle map, we use a box scanning algorithm to identify the regions where the largest changes in angle occur. Multiple regions may be detected and we retain the few largest clusters. Defects within those clusters can then be identified to belong to a string of defects separating two adjacent regions. Since the dislocations in one such cluster may not necessarily belong to one string (see the strings in Fig. 1b separating blue, red and green domains), we further need to segment the extracted strings into substrings. This is first based on the distances to the nearest dislocation neighbours of a dislocation and then refined by splitting strings further into substrings when distances and angles between dislocations exceed a certain threshold. This way, we obtain in one frame individual strings on each side of which we can probe the orientation of the adjacent region. Doing so for 151 frames in the sequence (from the 579 frames in the series studied here, clearly distinguishable grain boundaries only occur in frames 70 to 220) and taking strings with a length of at least three, we obtain the plot in Fig.  2a for the relative angle of adjacent domains versus the median distance of dislocations in one string. For comparison, we do the same for pairs of dislocations only. Filtering for longer strings greatly helps to reduce the scattering of the data, although in both cases the trend (smoothed curve) is similar. One can also notice a certain accumulation of data points at discrete distance values, reflecting the discrete nature of the Voronoi tessellation. Note that the algorithm works autonomously and is not supervised after the initial threshold parameters are set (e.g. for detecting changes in angle). A video illustrating the identification of defect strings, their extraction, and the probing of the adjacent orientations can be found in Additional file 2.

**Fig. 3:**
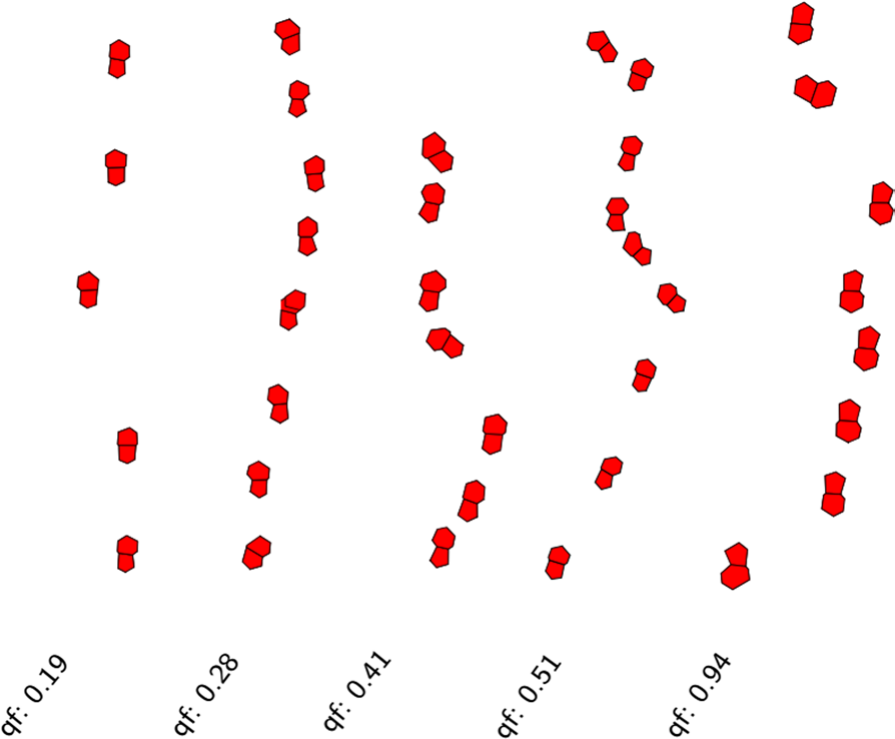
Attribution of a quality factor qf for the extracted defect pair strings based on how straight the lines are and on the angles between pairs of defect pairs. The lower the number the better

**Fig. 4:**
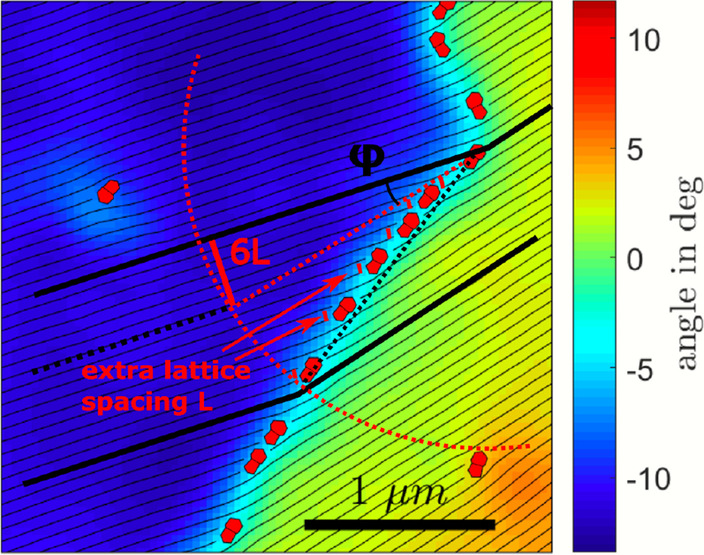
Illustration of the lattice splitting by a string of defects. The six defects in the string each add a lattice spacing *L*, causing a lattice splitting at an angle $\phi$

### Quality of Extracted Strings

We can further tweak our analysis by only looking at ‘high-quality’ strings, i.e. we would like to discard strings that comprise a significantly large variation in distances and angles between dislocations and generally deviate from a straight line. Based on these quality criteria, we attribute a ‘quality factor’ to each string—the lower this factor the better, as illustrated in Fig. 3.

The choice of strings of different lengths and quality considerably affects the variation of data points around their mean, as seen in Fig. 2b.

### Lattice Twisting Geometry

To explain our observations, we inspect Fig. 4. Introducing a dislocation in a lattice means squeezing in an additional lattice line. A high-density defect string results in many extra line spacings that can only be accommodated by a large twisting angle of the adjacent domains and vice versa for a low-density defect string. We see in the illustration how this would follow an arcsin relation between the relative angle of adjacent domains ($\phi$) and the ratio of the lattice constant *L* and the median distance between dislocations *D*:

$$\begin{aligned} \phi = \arcsin \left( \dfrac{L}{D}\right) \end{aligned}$$

where $6L/6D = L/D$ is used in the illustration. This relation is shown as the green curve in Fig. 2b, and we can see a very good agreement with our observations.

Recently, a study with a similar result has been published [23]. However, here we focus in more detail on the algorithm that we use to extract and quantify the dislocation lines. Furthermore, in contrast to [23] where the authors mention their algorithm fails to extract dislocation lines separating domains with a relative angle below 10 degrees, our algorithm does so just fine and lines can be extracted for the whole spectrum of observed twisting angles without the need for manual corrections. We used one minor simplification, which is to discard all non-paired defects, i.e. clusters of higher order. Our analysis could therefore be further refined by calculating the Burgers vector for these defect clusters to determine to what extent they behave as dislocations or not. Essentially though, discarding such clusters means producing a gap in a string of dislocations so that in such an instance one would consider two strings of dislocations instead of one. In practice, this means that the elimination of defect clusters has only a limited impact on our results. At the same time, they are often artefacts as mentioned above, stemming from point identification in blurry parts of a TEM image and therefore should be discarded anyway.

## Conclusion

In summary, we have developed an algorithm, which can extract lines of dislocations that separate adjacent domains of different orientations and classify them into substrings of different quality. With this kind of statistical probing of grain boundaries, we achieve the equivalent of systematically generating grain boundaries separating domains with gradually decreasing dislocation density. The resulting relationship between the median distance between dislocations and the relative angle between domains is a geometrical one: for a given twisting angle the average distance from one dislocation to the next is determined by the lattice spacing *L* and therefore follows an arcsin relation.

Finally, we show how the dynamical properties of skyrmion lattices and their observation in real space and real time by LTEM qualify skyrmion systems as testbeds for other condensed matter systems where real space observations are either not possible or similar statistics are not achievable.

## Supplementary Information

**Additional file 1.** More details on the skyrmion position identification routine.

**Additional file 2.** Video illustrating the defect string identification, their extraction and the probing of the orientations of the adjacent domains frame by frame.

## Data Availability

The datasets used and/or analysed during the current study are available from the corresponding author on reasonable request.

## Abbreviations

TEM: Transmission electron microscopy
LTEM: Lorentz transmission electron microscopy
FIB: Focused ion beam

## References

[1] Skyrme THR (1962). A unified field theory of mesons and baryons. Nucl Phys.

[2] Bogdanov AN, Yablonskii DA (1989) Thermodynamically stable “vortices” in magnetically ordered crystals. The mixed state of magnets. Zh Eksp Teor Fiz 95:178

[3] Mühlbauer S, Binz B, Jonietz F, Pfleiderer C, Rosch A, Neubauer A (2009). Skyrmion lattice in a chiral magnet. Science.

[4] Fert A, Cros V, Sampaio J (2013). Skyrmions on the track. Nat Nanotechnol.

[5] Garst M, Waizner J, Grundler D (2017). Collective spin excitations of helices and magnetic skyrmions: review and perspectives of magnonics in non-centrosymmetric magnets. J Phys D Appl Phys.

[6] Zhang X, Ezawa M, Zhou Y (2015). Magnetic skyrmion logic gates: conversion, duplication and merging of skyrmions. Sci Rep..

[7] Pinna D, Abreu Araujo F, Kim JV, Cros V, Querlioz D, Bessiere P (2018). Skyrmion gas manipulation for probabilistic computing. Phys Rev Appl.

[8] Romming N, Hanneken C, Menzel M, Bickel JE, Wolter B, von Bergmann K (2013). Writing and deleting single magnetic skyrmions. Science.

[9] Romming N, Kubetzka A, Hanneken C, von Bergmann K, Wiesendanger R (2015). Field-dependent size and shape of single magnetic skyrmions. Phys Rev Lett.

[10] Huang P, Schönenberger T, Cantoni M, Heinen L, Magrez A, Rosch A (2020). Melting of a skyrmion lattice to a skyrmion liquid via a hexatic phase. Nat Nanotechnol.

[11] Karube K, White JS, Reynolds N, Gavilano JL, Oike H, Kikkawa A (2016). Robust metastable skyrmions and their triangular-square lattice structural transition in a high-temperature chiral magnet. Nat Mater.

[12] Karube K, White JS, Morikawa D, Dewhurst CD, Cubitt R, Kikkawa A (2018). Disordered skyrmion phase stabilized by magnetic frustration in a chiral magnet. Sci Adv.

[13] White JS, Prša K, Huang P, Omrani AA, Živković I, Bartkowiak M (2014). Electric-field-induced skyrmion distortion and giant lattice rotation in the magnetoelectric insulator Cu_2_OSeO_3_. Phys Rev Lett.

[14] White JS, Živković I, Kruchkov AJ, Bartkowiak M, Magrez A, Rønnow HM (2018). Electric-field-driven topological phase switching and skyrmion-lattice metastability in magnetoelectric Cu_2_OSeO_3_. Phys Rev Appl.

[15] Crisanti M, Reynolds N, Živković I, Magrez A, Rønnow HM, Cubitt R (2020). In situ control of the helical and skyrmion phases in Cu_2_OSeO_3_ using high-pressure helium gas up to 5 kbar. Phys Rev B.

[16] Živković I, Pajić D, Ivek T, Berger H (2012). Two-step transition in a magnetoelectric ferrimagnet Cu_2_OSeO_3_. Phys Rev B.

[17] Levatić I, Šurija V, Berger H, Živković I (2014). Dissipation processes in the insulating skyrmion compound Cu_2_OSeO_3_. Phys Rev B.

[18] Levatić I, Popčević P, Šurija V, Kruchkov A, Berger H, Magrez A (2016). Dramatic pressure-driven enhancement of bulk skyrmion stability. Sci Rep.

[19] Seki S, Ishiwata S, Tokura Y (2012). Magnetoelectric nature of skyrmions in a chiral magnetic insulator Cu_2_OSeO_3_. Phys Rev B.

[20] Rajeswari J, Huang P, Mancini GF, Murooka Y, Latychevskaia T, McGrouther D (2015). Filming the formation and fluctuation of skyrmion domains by cryo-Lorentz transmission electron microscopy. Proc Natl Acad Sci.

[21] Kruchkov AJ, White JS, Bartkowiak M, Zivkovic I, Magrez A, Rønnow HM (2018). Direct electric field control of the skyrmion phase in a magnetoelectric insulator. Sci Rep.

[22] Huang P, Cantoni M, Kruchkov A, Rajeswari J, Magrez A, Carbone F (2018). In situ electric field skyrmion creation in magnetoelectric Cu_2_OSeO_3_. Nano Lett.

[23] Pöllath S, Wild J, Heinen L, Meier TNG, Kronseder M, Tutsch L (2017). Dynamical defects in rotating magnetic skyrmion lattices. Phys Rev Lett.

