# Supplementary material for: Direct Visualisation of Skyrmion Lattice Defect Alignment at Grain Boundaries
Source: Nanoscale Res Lett. 2022 Jan 28;17:20. doi: 10.1186/s11671-022-03654-y (PMC8799828; doi:10.1186/s11671-022-03654-y)
Supplement: Supplementary file 1 — Additional file 1. More details on the skyrmion position identification routine. [file 11671_2022_3654_MOESM1_ESM.pdf]

**SUPPLEMENTARY INFORMATION**

# Direct Visualisation of Skyrmion Lattice Defect Alignment at Grain Boundaries

Thomas Schönenberger[1*], Ping Huang[2], Lawrence D. Brun[1], Li Guanghao[1], Arnaud Magrez[1], Fabrizio Carbone[1] and Henrik M. Rønnow[1]

[*]Correspondence:
thomas.schoenenberger@epfl.ch
[1]Laboratory for Quantum Magnetism (LQM), Institute of Physics, École Polytechnique Fédérale de Lausanne (EPFL), Lausanne, Switzerland
Full list of author information is available at the end of the article

## Details on Defect Line Extraction

The steps in the identifcation of skyrmions and the extraction of defect lines are illustrated using frame 150 from the series. The raw TEM image is shown in figure 1. The application of an log-type filter enhances the contrast and smoothes out contrast variations, see figure 2. This is the basis for the identification of points, where local minima can in principle be directly identified to be sykrmion positions, see figure 3, where the identified positions (red dots) are plotted on the raw image seen in figure 1. The voronoi diagram as introduced in the paper and based on these identified points is shown in figure 4. The vertices of each polygon are used to calculate the orientation for each skyrmion. Due to the hexagonal symmetry of the lattice these angles are between -15° and 15°, the result is shown in figure 5. With a box scanning algorithm we can extract the regions of largest change in the angle map, from which we can extract the defect lines, see figure 6. We divide those extracted strings into substrings as outline in the main text. On both sides of these substrings the orientation of the adjacent grain boundaries can be probed, as illustrated in figure 7. We further illustrate the procedure of extracting grain boundaries across the series by making a video, to be also found in the Supplementary Information. Each frame of the video consists of the extracted grain boundary on the left (green), the angle map along with the regions where the adjacent orientations are probed (center) and the plot of the relationship of the separation angle of orientations at the grain boundary vs the mean distance within the defect line at that grain boundary, as shown in figure 8. Each frame in the video adds one or more points in the plot.

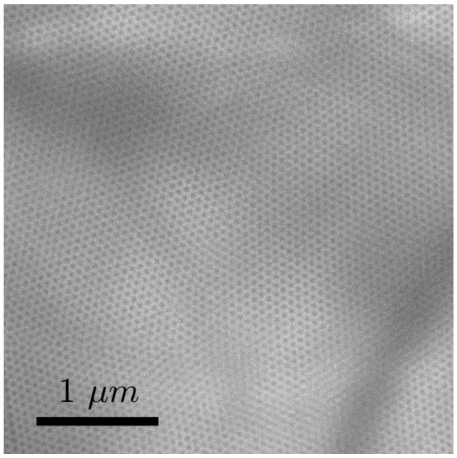


Figure 1: Raw TEM image (frame 150).

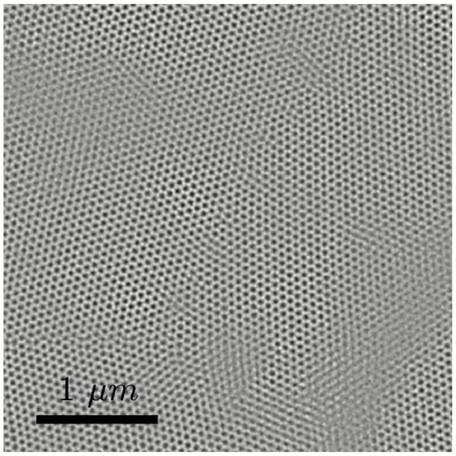


Figure 2: TEM image after application of log-type filter.

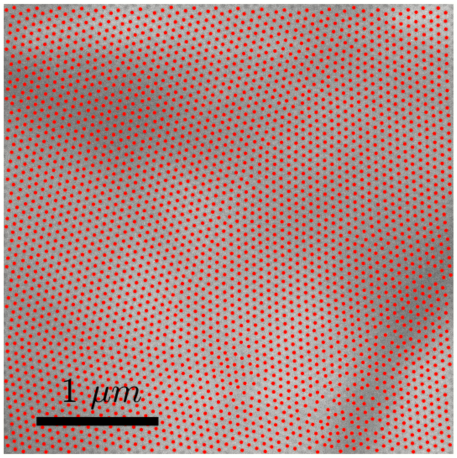


Figure 3: Identification of local minima as skyrmion positions (red dots) on raw TEM image.

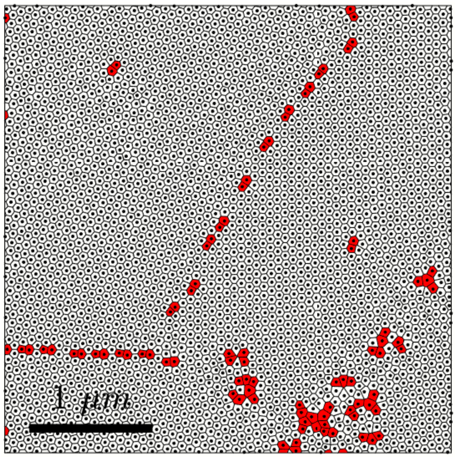


Figure 4: Voronoi diagram of the skyrmion positions as extracted from the image.

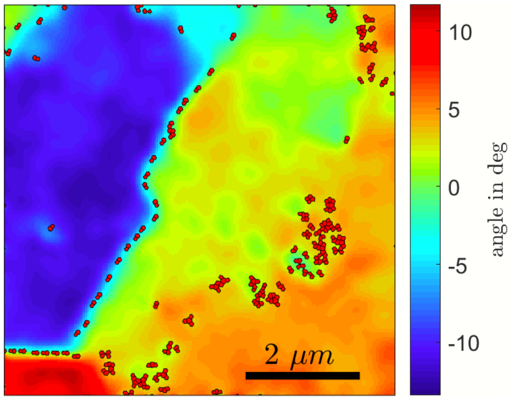


Figure 5: Angle map revealing the different lattice domains.

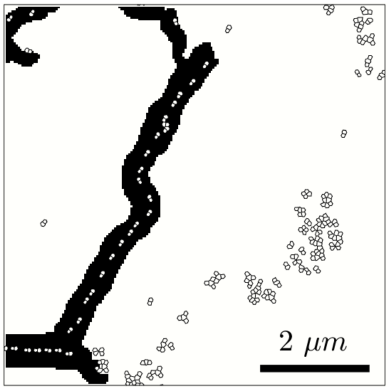


Figure 6: Based on the angle map, regions of large angular change are extracted.

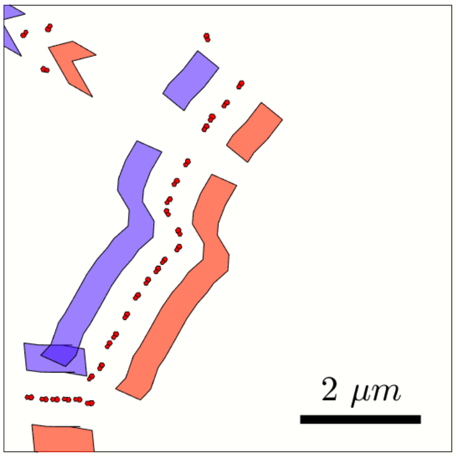


Figure 7: Probing the orientation to both sides of the extracted defect strings.

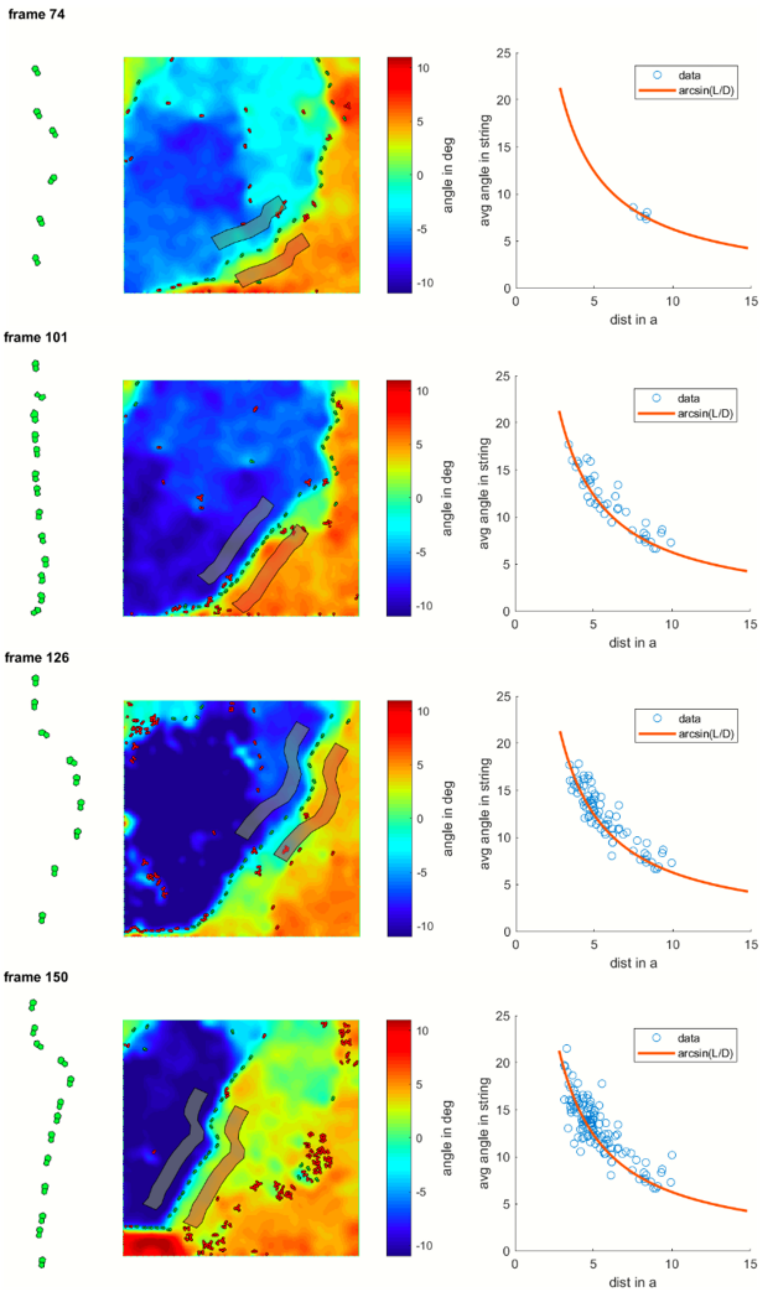


Figure 8: Frames from the video illustrating the grain boundary extraction.

**Author details**
[1]Laboratory for Quantum Magnetism (LQM), Institute of Physics, École Polytechnique Fédérale de Lausanne (EPFL), Lausanne, Switzerland. [2]State Key Laboratory for Mechanical Behavior of Materials, Xi'an Jiaotong University, Xi'an, China.

**References**
